# Paraffine

**Published:** 1860-06

**Authors:** R. Peckover


### PARAFFINE.

PARIS, May 22, 1860.

MESSRS. EDITORS :—In reply to many letters which I have received concerning my preparation of Paraffine, I would state that it can be used in taking impressions in all cases in which Gutta Percha is admissible ; but I need not say that it must be done carefully. The preparation must be warmed over a fire very gradually till soft enough, and the impression taken in the usual manner, care being taken to pass the finger over the surplus wax in the cup while in the mouth. After leaving the mouth, it should be plunged into cold water, and the plaster poured in. In vulcanite work it may be used instead of the Gutta Percha, or impression gum, by taking a sheet of the preparation of the requisite thickness, warming it and carefully pressing it on to the cast. You may then trim the labial sides to any required shape, paring it as thin as paper ; if necessary, dip it in cold water, and then proceed to fit in the mouth ; if it does not fit as well as you wish, you can press it so as to make it do so, and then pour plaster into it to suit your own views.

I throw these hints together that the profession may try the article, and if they like it on trial, use it—if not, throw it into their soldering lamp and burn it, for which it will answer as well as oil.

I am, gentlemen, your ob't serv't, R. PECKOVER.

The article can be obtained of Jno. T. Toland.

